# A new paper-based approach for teaching Bragg’s law

**DOI:** 10.1107/S2056989025010576

**Published:** 2025-11-28

**Authors:** Thomas E. Weirich

**Affiliations:** a RWTH Aachen University, Gemeinschaftslabor für Elektronenmikroskopie (GFE), Ahornstr. 55, D-52074 Aachen, Germany; Harvard University, USA

**Keywords:** Bragg’s law, X-ray diffraction, teaching, triangular ruler, crystal structure

## Abstract

A simple paper-based model is proposed, which is based on the right-angle condition for constructive inter­ference of Bragg’s law. As demonstrated, the model allows visualization and basic qu­anti­fication of the geometric relationships between wavelength, lattice spacing, and diffraction angle. Moreover, it facilitates the exploration of the limiting conditions for diffraction, angular dispersion, and the role of wavelength as a factor affecting crystallographic resolution.

## Abstract 2

A paper-based virtual X-ray diffraction experiment, which is based on the right-angle condition for constructive inter­ference, allows the implications of Bragg’s law to be studies in a simple way.

## Introduction

It is widely recognized that determining the atomic structure of matter is the key that allows science to develop a deeper understanding of the correlation between structure and the physical and/or (bio-)chemical properties of substances and materials. Consequently, structural analysis techniques always play a central role in the discovery and exploration of matter in many scientific disciplines. Despite their significance, the complexity of various structural analysis methods frequently presents challenges in explaining their underlying principles to non-specialists and students. To address this situation, several educational and outreach activities have been launched within the crystallographic community in recent years [see, for example, Abrahams *et al.* (2023 ▸), Bou-Nader *et al.* (2025 ▸), Irmer (2025 ▸) and references therein]. In this context, it is needless to mention, that understanding Bragg’s renowned law of X-ray diffraction is of fundamental importance. However, the author’s teaching experience has shown that students often focus very much on just reciting the formula of Bragg’s law, but find it more difficult to sketch the corresponding geometry for deriving conclusions for real experiments. This phenomenon may also be associated with the extensive use of tablet computers, as students now rarely use paper and pencil. To resolve this pedagogical issue, an alternative approach has been developed and tested for some years within various lectures on scattering methods for condensed matter research for masters students of applied geosciences, materials sciences and physics. The approach proposed here can be considered as a virtual X-ray diffraction experiment offering students a hands-on, paper-based exploration of the geometric principles of Bragg’s law. This virtual experiment requires minimal preparation time and no special equipment. It only requires a sheet of paper, a pencil and a triangular ruler, making it easy to implement in a variety of educational settings. As the model relies only on simple geometric constructions, such as lengths and right angles, and does not require the explicit use of trigonometric functions, it could possibly also be adapted for use outside of university teaching, once students have gained a basic understanding of wave inter­ference and light reflection. However, its effectiveness in these contexts remains to be explored.

## Background

**Figure 1:**
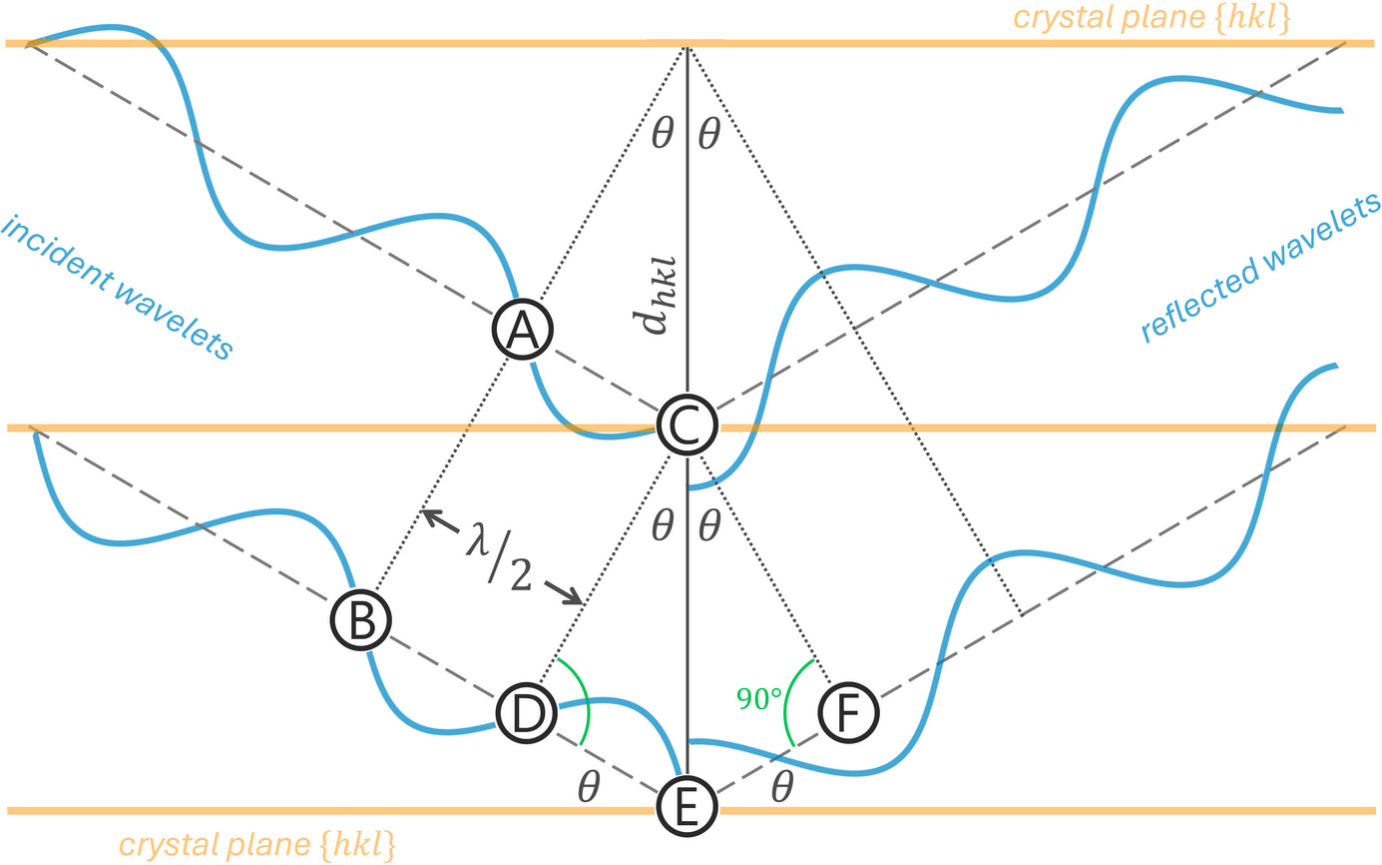
Simplified geometric model to illustrate Bragg diffraction of parallel, monochromatic X-rays on the lattice planes {*hkl*} of a crystal. To generate an intensity maximum in the far field *via* constructive inter­ference, the reflected wavelets that emerge from the two lattice planes at C and E, need to be in phase. According to the kinematic theory and the Born approximation, the two reflected waves are shifted by −π/2 with respect to the incident wavelets as shown (Cowley, 1975 ▸). Under the condition that the incident wavelets at A–B and C–D are in phase, this can only be achieved if the path difference D–E–F between the wavelet emerging from the bottom lattice plane at E and the wavelet from lattice plane at C is an integral multiple of the wavelength according to Equation (1). Note, that the Bragg angle θ appears not only as the glancing angle, but also in the two right-angled triangles cornered by C–D–E and C–E–F. Moreover, it is important to note that the opposite minor cathetus in these right-angled triangles has the length λ/2, which is one of the key parameters that is going to be changed within this teaching concept.

The diffraction of X-rays by crystals was first discovered in 1912 by Walter Friedrich, Paul Knipping, and Max von Laue. This groundbreaking discovery gave rise to some major analytical techniques that are now routinely used in thousands of laboratories worldwide. These methods are X-ray diffraction (XRD), wavelength-dispersive X-ray spectroscopy (WDXS or WDS), and X-ray fluorescence (XRF). All of these methods have become indispensable tools for a wide range of analytical applications, which are based on the fundamental law of diffraction, first formulated by William Lawrence Bragg in 1913 [for the history of the discovery of X-ray diffraction and the elaboration of Bragg’s law see, for example, Bijvoet *et al.* (1969 ▸) and Authier (2013 ▸)]. As outlined in his Nobel lecture (Bragg, 1922 ▸), W. L. Bragg inter­preted the phenomenon of X-ray diffraction by assuming that a monochromatic beam of X-rays falls on a regular array of atoms in a crystal that are lying on (virtual) parallel and equally spaced lattice planes within the crystal structure. This approach allowed him to treat X-ray diffraction as the reflection of waves from these lattice planes that satisfy the condition 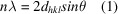 where *n* is an integral multiple of the wavelength λ of the used X-rays, *d_hkl_* the lattice spacing (inter­planar distance) between adjacent lattice planes on the basis of a set of *hkl* indices and θ the Bragg angle (‘glancing’ angle) at which an X-ray reflection maximum can be observed. A corresponding geometrical model for the standard case of an incident beam of parallel monochromatic X-rays (*i.e. n* = 1) of wavelength λ is shown in Fig. 1 ▸. For an inter­active online version that allows the parameters to be adjusted, the inter­ested reader is referred to Bragg’s Law Simulator (https://sg.iwant2study.org/ospsg/index.php/inter­active-resources/physics/06-modern-physics/02-nuclear/709-braggslaw) or the Wolfram Demonstrations Project (https://demonstrations.wolfram.com/BraggsLaw/). It is important to bear in mind, that the model shown in Fig. 1 ▸ is a great simplification of the real physical process, since, in reality, the lattice planes shown in the illustration are populated by a two-dimensional array of regularly arranged atoms situated nearby or exactly on the planes, such as in simple structures like NaCl or CsCl. When an incoming X-ray photon inter­acts with the electron cloud of an atom in this array, each atom starts to behave like a tiny X-ray source that emits radiation with the same wavelength as the incoming beam. This process is called Thomson scattering (Cowley, 1975 ▸).

Because the atoms are arranged regularly on a lattice, the scattered X-rays combine and form a common wavefront according to Huygens’ principle (Bragg, 1949 ▸). Unlike visible light, X-rays can penetrate solid matter deeply, and also create wavefronts on sets of equidistant lattice planes within the crystal. These wavefronts are usually out of phase with one another. However, as illustrated in Fig. 1 ▸ (and later in Fig. 5), there is always a specific angle of incidence, known as the Bragg angle θ, for which the X-rays scattered from different lattice planes are in phase. Consequently, an intensity maximum, termed the X-ray reflection, is generated by constructive inter­ference in the far field. It is therefore necessary to consider at least two parallel lattice planes for the modelling of this inter­ference effect, as shown in Fig. 1 ▸. Similarly, when visible light is reflected by a mirror, the angle of incidence is equal to the angle of reflection under these conditions. However, although this virtual experiment just focuses on the exact Bragg angle, where all diffracted rays are perfect in phase with each other, it should be noted, that a small deviation from the exact Bragg angle will not immediately result in complete destructive inter­ference of the diffracted rays. In fact, the intensity of a reflection in the far field can be modelled mathematically using a *Sinc* [(sin *x*)/*x*] function. This function decreases quickly, but then oscillates with a much smaller and increasingly lower amplitude as the deviation from the Bragg angle increases. This behaviour is also known as the ‘rocking curve’ of a reflection (Cowley, 1975 ▸).

## Exploring the geometry of Bragg’s law with a triangular ruler

**Figure 2:**
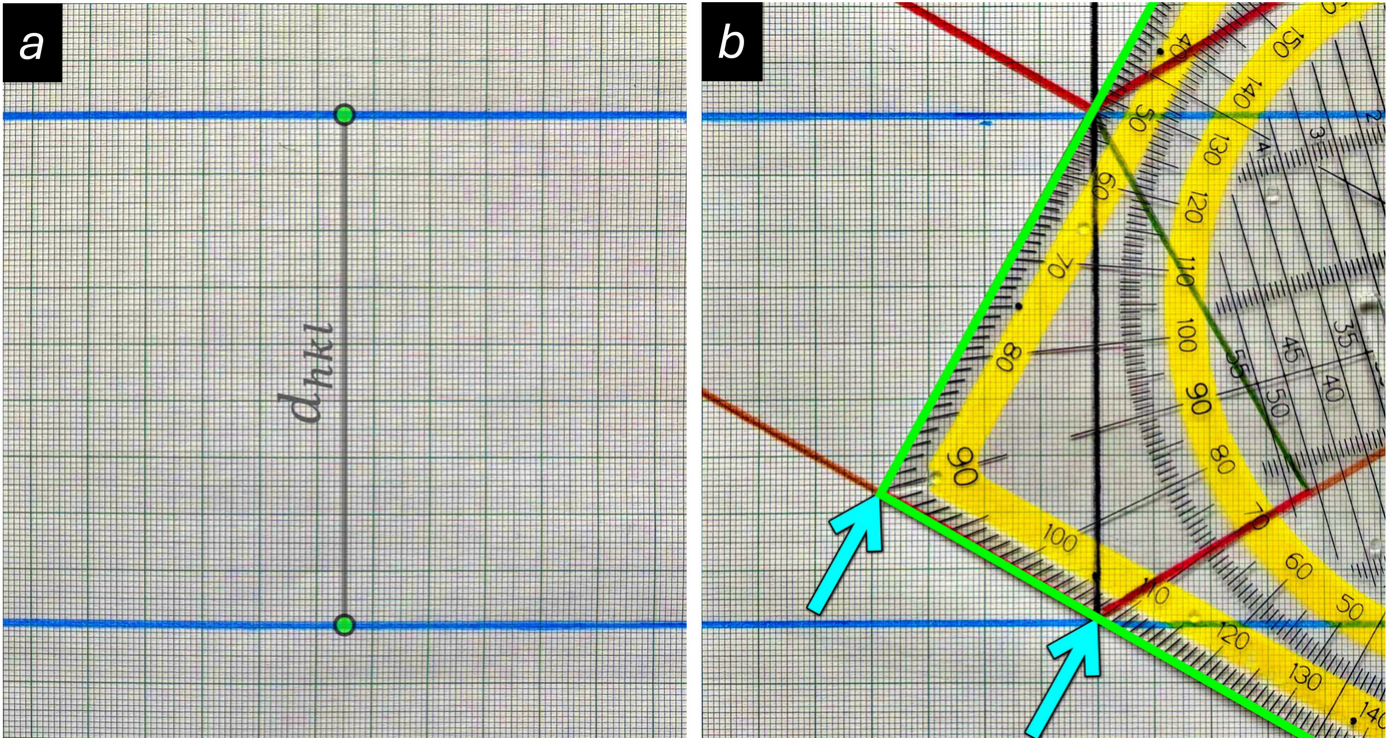
(*a*) In order to set up the hands-on experiment, two parallel horizontal lines representing a set of {*hkl*} lattice planes must be drawn. The distance between these lines defines the lattice spacing *d_hkl_*. The two green spots at the ends of the line defining the lattice spacing mark the anchor points that the edges of the triangle ruler must cross to comply with the required condition for constructive inter­ference. (*b*) Here a standard German triangular ruler (*Geodreieck*) has been adjusted to fit the lower left triangle of the geometric construction. Note that the green edges of the triangle cross the anchor points marked in Fig. 2 ▸*a*. It is also important to note that the distance on the lower edge of the triangular ruler between the two arrows defines the half wavelength λ/2. Thus, by shortening or lengthening this distance along the lower edge, different wavelengths can be defined for the virtual X-ray diffraction experiment. Note added on manuscript revision: one reviewer suggested using a needle or pin inserted into the anchor points to maintain contact with these points when exploring changes in wavelength and diffraction angle.

The following description of the hands-on virtual X-ray experiment is based exclusively on the geometry of the right-angled triangles shown in Fig. 1 ▸, because these triangles bear the conditions for the required in-phase scattering of the wavelets that emerge from the different lattice planes. For setting up a virtual X-ray experiment at least two parallel horizontal lines are drawn on a sheet of letter or A4-sized paper. These lines represent the lattice planes of the crystal in the virtual X-ray experiment. In the example in Fig. 2 ▸*a*, the lattice planes are separated by a lattice spacing *d_hkl_* of 9 cm. Now that the lattice planes of the crystal have been defined, the next step is to choose the wavelength for the virtual diffraction experiment. For this, the triangular ruler will be used. As shown in Fig. 2 ▸*b*, the length of the lower edge between the two arrows (measured from the corner) defines half the wavelength. Shortening or lengthening this distance by the same value on both edges thus sets different wavelengths for the virtual X-ray diffraction experiment. [Note that the standard German triangle ruler (*Geodreieck*) has a degree scale along these edges, so the selected value can easily be converted to metric measurements by drawing the corresponding length on paper and measuring it with the ruler. However, any other type of geometric triangle can also be used. Corresponding distances marked with a pencil can again be measured afterwards using a normal ruler.] The Bragg angle for each selected wavelength and lattice spacing can then easily determined by aligning the lower anchor point with the half wavelength distance on the lower edge of the triangle ruler while the upper edge of the triangle ruler passes through the upper anchor point (see Fig. 2 ▸*b*). This simple geometrical restriction is everything that is needed for studying the various settings in this virtual X-ray diffraction course.

### Virtual experiments with varying wavelength

**Figure 3:**
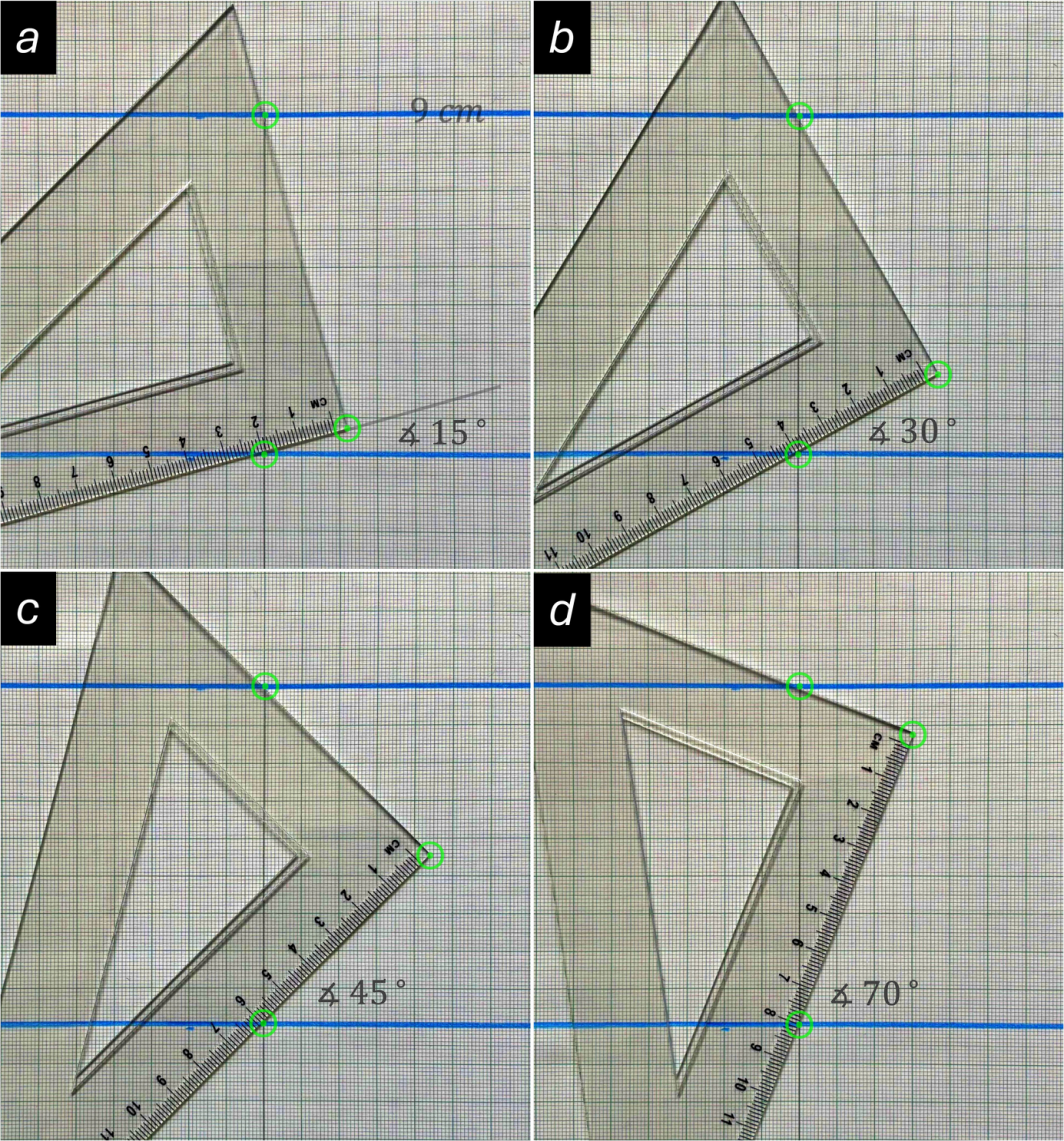
By applying the geometrical constraints for constructive inter­ference introduced in Fig. 2 ▸*a*, it can be shown that increasing the wavelength (at the scale of the triangular ruler) leads to an increase in the Bragg angle θ (the values given here for θ were measured using a triangular ruler). Note that the limit for diffraction is reached at θ = 90°, when λ/2 becomes the same as the lattice spacing. Any wavelength larger than λ/2 means that the edges of the ruler cannot be aligned with the anchor points on the lattice planes. For a real diffraction experiment, this implies that no *d*-spacing smaller than λ/2 can be determined, which ultimately affects the resolution of the data (see main text).

First insights into Bragg’s law are easily gained by trying out different X-ray wavelengths, which is something that is recommended to be done first. In the examples in Fig. 3 ▸, four different setups with increasing wavelengths are shown. Without the need for analysing the formula in Equation (1) or calculating any values, one can see immediately that increasing the wavelength on the triangular ruler leads to an increase of the Bragg angle θ. Furthermore, for a given lattice plane distance the limiting wavelength for diffraction is easily determined. This limit is reached at θ = 90° for λ/2 equal to *d_hkl_*, because any larger value for λ/2 would make it impossible to match the edges of the triangle ruler simultaneously with the anchor points on the lattice planes. In this context, it should be realised, that every reflection with a diffraction angle larger than 45° (2θ > 90°) corresponds to a reflection that is diffracted backwards (towards the direction of the incident primary X-ray beam). Since the maximum diffraction angle can never exceed 180°, again θ = 90° (sin θ = 1) is the maximum angle. Although this is beyond the scope of the present article, it is worth noting that the reciprocal value of the λ/2 limit identified here corresponds to the diameter of the Ewald sphere, which is also known as the sphere of reflection in reciprocal space (Ewald, 1921 ▸). However, to make things more practical, this approach can now be employed to estimate the largest wavelength that can be used for diffraction on a given lattice spacing when the instrument is limited in its 2θ measuring range. Since the maximum collection range of X-ray powder diffractometers is usually 2θ ≈ 150°, the crystallographic resolution (*i.e.* the smallest measurable *d* value) can be determined geometrically as described in the following. In order to make the measurements with the ruler from the drawing sufficiently accurate, it is recommended to use for this two parallel lines that are at least 9 cm apart. Then, starting from the lower anchor point, a line with the limiting Bragg angle θ of 75° is drawn until the upper lattice plane is reached. The triangular ruler is now moved along the drawn line to the point where a perpendicular line can be drawn that inter­sects with the upper anchor point. Measurement of the lengths that correspond to λ/2 and *d_hkl_* with the ruler allows the ratio in Equation (2) to be calculated. By use of this rearranged Bragg equation, it is possible to carry out some simple real-case calculations.
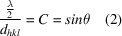
In numbers: For a model with *d_hkl_* = 10 cm, and a limiting Bragg angle of 75°, the length of the largest possible half wavelength is about 9.6 cm, which yields a value of 0.96 for *C* in Equation (2). If the required smallest *d*-spacing in a real-world experiment is 0.8 Å, and the instrument limitations are the same as above, the value for the largest wavelength is given by 2· (0.96 · 0.8 Å) ≈ 1.54 Å, which is almost the exact value of Cu *K*_α_X-rays.

Conversely, if the smallest available *d*-spacing is requested for Cr *K*_α_X-rays (λ ≈ 2.29 Å) using the same X-ray powder diffractometer, the answer is (0.5 · 2.29 Å)/0.96 = 1.19 Å. Why is it important to know this value? For data from single-crystal diffraction, the minimum separation at which two atoms can be distinguished (the limit of resolution *LR*) is given by Equation (3) (Stenkamp & Jensen, 1984 ▸).
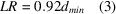
Thus, the smaller the measured *d*-values, the better the atoms in a crystal structure can be distinguished from one another and the more precisely the structure can be determined.

### Virtual experiments with varying lattice spacings

**Figure 4:**
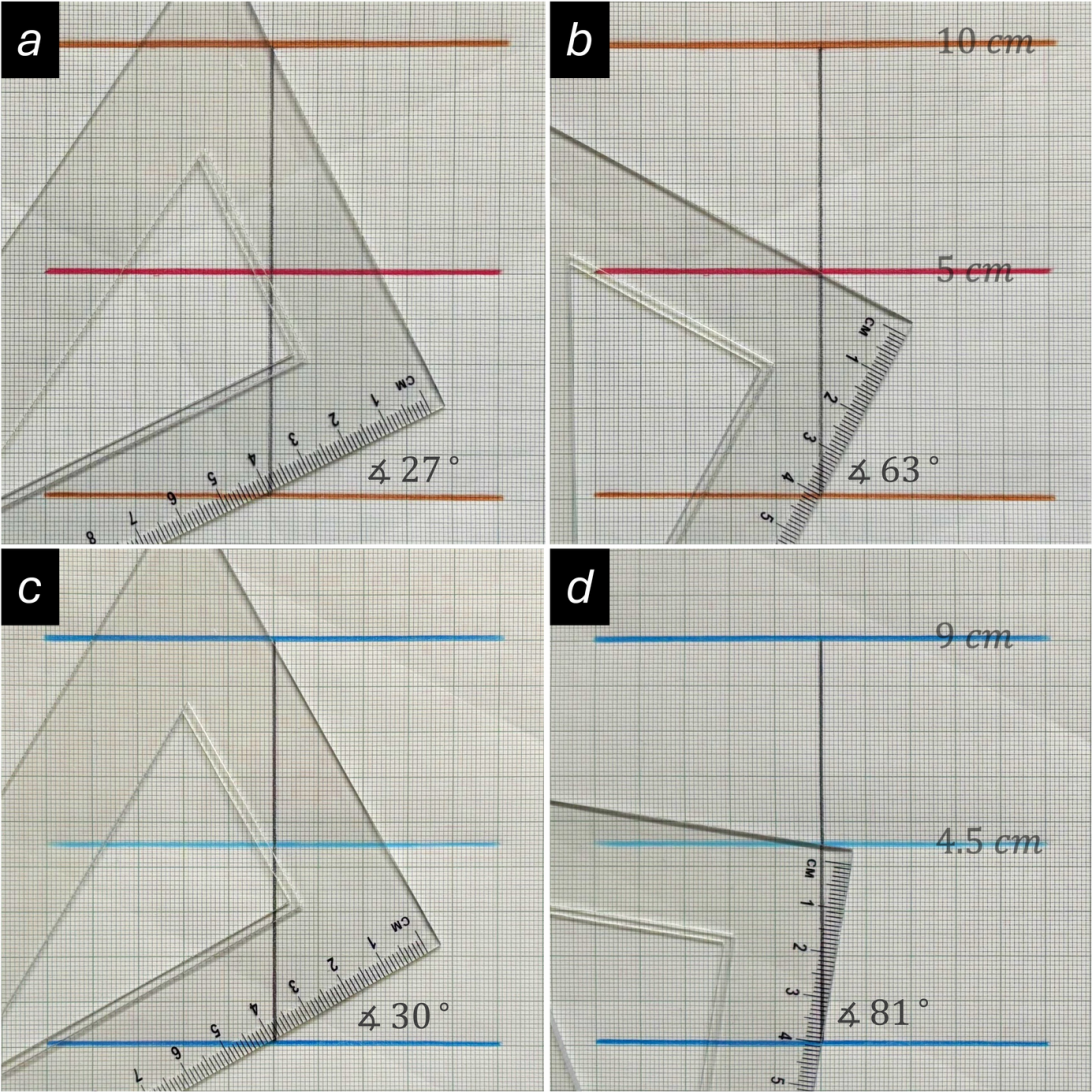
A set of virtual constant wavelength experiments on two sets of lattice spacings. As shown in Fig. 4 ▸*a* and 4b, the lattice spacings of the first model are 10 cm and 5 cm, respectively. The other model consists of inter­planar spacings of 9 cm and 4.5 cm, as illustrated in Fig. 4 ▸*c* and 4*d*. Each set represents the base lattice spacing (*h* = 1) and its higher order (*h* = 2). A comparison of the results in Fig. 4 ▸*a* and 4*c*, and Fig. 4 ▸*b* and 4*d*, shows that the angular dispersion is about ten times smaller for larger lattice spacings than for smaller ones (see main text). Consequently, measuring Bragg angles and calculating values using X-ray reflections at higher diffraction angles produces more precise results with smaller errors.

Aside from the wavelength, the other variable parameter in Equation (1) is the lattice spacing. It will now be demonstrated how this geometrical approach can be used to study the dependence of the attainable precision as a function of the Bragg angle. This will be achieved by conducting virtual experiments on two sets of lattice spacings using a constant wavelength. As shown in Fig. 4 ▸*a* and 4*b*, the lattice spacings of the first model are 10 cm and 5 cm, respectively. The other model consists of inter­planar spacings of 9 cm and 4.5 cm, as illustrated in Fig. 4 ▸*c* and 4*d*. Thus, each set represents the base lattice spacing (*h* = 1) and its higher order (*h* = 2), which is half the distance of the base lattice spacing. The half wavelength as measured on the ruler is about 4.4 cm. As shown in Fig. 4 ▸*a* and 4*c*, the corresponding measured Bragg angles from the geometric constructions are about 27 and 30° for the larger lattice spacings, and 63 and 81° for the higher order ones. As the reader may have noticed, there are some deviations between the measured angles and the numerically calculated values. These differences are caused by inaccuracies in the drawings and the measurements with the triangular ruler. However, these small deviations are not of importance in the present context, as the primary aim is to provide a basic understanding of the relationships rather than to determine exact values by this method. If the angular dispersion defined as Δθ/Δ*d_hkl_* is calculated, we obtain 3°/Å for the two larger lattice spacings and 36°/Å for the two smaller lattice spacings. For comparison, the corresponding calculated values using Equation (2) are 3.23°/Å and 32.6°/Å, respectively. These values show that the rate of change of the Bragg angle with respect to the lattice spacing (the ‘sensitivity’) is approximately ten times higher for the set of smaller lattice spacings than for the larger ones. This has also significant implications for real experiments, as slight differences in the lattice spacings will be much easier to detect at larger Bragg angles, due to the higher angular dispersion for small lattice distances *d_hkl_*. Thus, for example, a small tetra­gonal distortion of a cubic lattice may not be resolved at lower Bragg angles but will become apparent at higher angles. Moreover, the higher angular dispersion, and therefore better precision, at larger diffraction angles is also the reason why high-angle (high-order) reflections are commonly used for lattice-parameter refinement in single crystal X-ray diffraction.

### Discussion

The model proposed here for virtual X-ray diffraction experiments allows systematic investigation of how wavelength affects the Bragg angle, attainable resolution, and angular dispersion, which are some of the key aspects of X-ray diffraction analysis. Beyond its conceptual simplicity, the virtual X-ray diffraction experiment also promotes real problem-solving skills, as it is based on manual graphical thinking that highlights the concept of *learning through drawing* (Ainsworth & Scheiter, 2021 ▸) rather than a numerical and calculation-based approach. Another advantage of the here proposed geometric approach is that it enables exploration of the implications of Bragg’s law without the need for its formula. This will also encourage students who find formulas too abstract to engage with it.

## Conclusions and outlook

**Figure 5:**
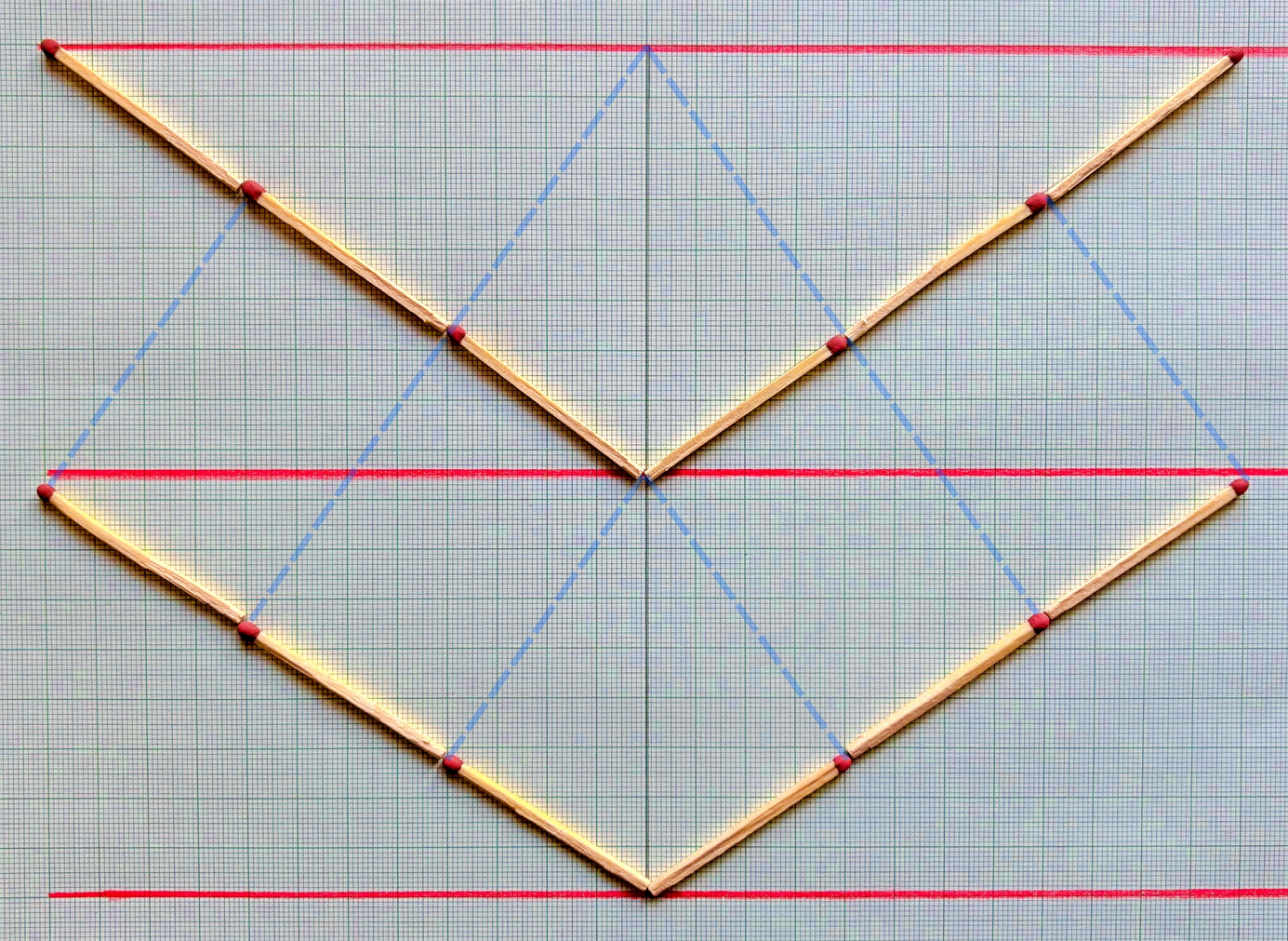
An alternative to drawing is to use matches to study the geometry of Bragg’s law. Here, the length of a match corresponds to half the wavelength of the used X-rays. In this case the goal for the participants is to find an arrangement of the matches where the imaginary dashed blue lines, which link the upper anchor point with the head of the match, make a right angle. This puzzle is a very useful method to understand Bragg’s law that for a given matchstick length (λ/2) and lattice spacing, there is always only one configuration that will satisfy the required right-angle condition.

A simple, paper-based virtual X-ray diffraction experiment is proposed that enables students to explore the geometry and implications of Bragg’s law through hands-on geometric construction and measurement. The proposed model thus offers an alternative to the typically formula based inter­pretation of Bragg’s law in teaching, that perhaps is more stimulating, as it requires the student activity. As the proposed method requires only basic materials, it has possibly also some potential to be used in educational settings outside university teaching, for which it was originally developed by the present author. As an example for a modified version outside university teaching, the model could be slightly adapted and matches could be used instead of a triangular ruler. So, the learners can also explore the restraints of Bragg’s law through a more tactile perception, as illustrated in Fig. 5 ▸.

## Comment on the tools used

Different triangular rulers have been used to prepare the figures shown in this article. In principle, it does not matter whether a set square (British) / triangle (American), like in Figs. 3 ▸ and 4 ▸, or a *Geodreieck* (German), as in Fig. 2 ▸, is used, so long it provides a fixed right angle. Thus, even a makeshift rectangle made from a folded cardboard in combination with a standard ruler could be used for the exercises suggested here.
